# Vascular Reactivity Concerning *Orthosiphon stamineus* Benth-Mediated Antihypertensive in Aortic Rings of Spontaneously Hypertensive Rats

**DOI:** 10.1155/2013/456852

**Published:** 2013-06-26

**Authors:** Nurul Maizan Manshor, Aidiahmad Dewa, Mohd Zaini Asmawi, Zhari Ismail, Nadiah Razali, Zurina Hassan

**Affiliations:** ^1^Department of Physiology, School of Pharmaceutical Sciences, Universiti Sains Malaysia, 11800 Minden, Penang, Malaysia; ^2^Department of Pharmacology, School of Pharmaceutical Sciences, Universiti Sains Malaysia, 11800 Minden, Penang, Malaysia; ^3^Department of Chemistry, School of Pharmaceutical Sciences, Universiti Sains Malaysia, 11800 Minden, Penang, Malaysia; ^4^Centre for Drug Research, Universiti Sains Malaysia, 11800 Minden, Penang, Malaysia

## Abstract

*Orthosiphon stamineus* Benth has been traditionally used to treat hypertension. The study aimed to investigate the vascular reactivity of water extract (WOS) and water : methanolic (1 : 1) extract (WMOS) of *Orthosiphon stamineus* Benth and AT_1_ receptors blocker in the mechanisms of antihypertensive mediated by **α**_1_-adrenergic receptor and EDNO and PGI_2_ releases in the SHR aortic rings. SHR (230–280 g) were divided into four groups: control, WOS, WMOS, and losartan. After being fed orally for 14 days, the aorta was harvested and subjected to PE (10^−9^ to 10^−5^ M) and ACh (10^−9^ to 10^−5^ M) with and without L-NAME (100 *µ*M) and indomethacin (10 *µ*M), respectively. WOS, WMOS, and losartan significantly reduced the contractile responses to PE intact suggesting the importance of endothelium in vasorelaxation. Losartan significantly enhanced the ACh-induced vasorelaxation. L-NAME significantly inhibited the ACh-induced relaxation in all groups. Indomethacin enhanced ACh-induced vasorelaxation in WMOS. Collectively, *Orthosiphon stamineus* leaves extract reduced vasoconstriction responses by the alteration of **α**_1_-adrenergic and AT_1_ receptors activities. The involvement of EDNO releases was clearly observed in this plant. In WOS, PGI_2_ releases might not participate in the ACh-induced vasorelaxation. However, in WMOS, enhancement of vasorelaxation possibly due to continuous release of PGI_2_.

## 1. Introduction

*Orthosiphon stamineus *Benth (syn.: *O. aristatus* (Bl.) Miq., *O. grandiflorus* Bold., *O. spicatus *(Thumb) Bak.; Lamiaceae) [1], or locally known as “Misai Kucing,” leaves extracts have been used as traditional medicine [2] and possess benefits such as antidiabetic, ability to increase plasma triglyceride and plasma HDL-cholesterol concentrations [3], anti-lithiatic and hypouricemic effects [4, 5], antifungal [6], and ability to treat kidney stone and urinary tract diseases [7–9]. It has traditionally been used in Java for the treatment of hypertension and diabetes [1]. Hypertension has been reported to be associated with endothelium dysfunction in both human and animal studies [10]. Endothelium regulates vascular tone by releasing vasoconstrictors such as endothelins, prostanoids and oxygen reactive species, and vasodilators such as nitric oxide (NO), prostacyclin (PGI_2_), and endothelial hyperpolarizing factor (EDHF). These vasodilators were a great discovery by Furchgott and Zawadzki in 1980 [11], known as endothelium derived relaxing factors (EDRF). It has been reported by Peach et al. [12] that releases of EDRF caused vasorelaxant effects of acetylcholine (ACh), which is dependent on the presence of the endothelial cells [11, 13]. 

Phenylephrine (PE) is a selective *α*_1_-adrenergic receptor agonist that increases arterial blood pressure by peripheral vasoconstriction. *α*_1_-Adrenergic receptors which exist postsynaptically are G-protein-coupled receptors, and thus activation of cellular signaling is subsequent to the interaction with a G-protein. Activation of these receptors on vascular smooth muscle leads to vasoconstriction. PE has predominantly *α*_1_-postjunctional receptors in rat's aorta [14]. Since PE is a selective *α*_1_-adrenergic receptor agonist and losartan is AT_1_ receptor blocker, there is possible relationship between AT_1_ and *α*_1_ receptors [15]. In addition, crosstalk between AT_1_ and *α*_1_ receptors in the smooth muscle of rabbit aorta is endothelium dependent [16]. It has been reported that MRC A isolated from *Orthosiphon stamineus* causes continuous decreases in systolic blood pressure (SBP) and heart rate (HR) after subcutaneous administration in conscious SHR [17]. However, studies on the antihypertensive mechanisms by *Orthosiphon stamineus *still remain unclear. The present study aimed to investigate the vascular reactivity of water extract (WOS) and water : methanolic (1 : 1) extract (WMOS) of *Orthosiphon stamineus *Benth and AT_1_ receptors blocker in the mechanisms of antihypertensive effects mediated by *α*_1_-adrenergic receptor and prostacyclin (PGI_2_) and endothelium-derived nitric oxide (EDNO) releases in the SHR aortic rings.

## 2. Materials and Methods

### 2.1. Preparation of *Orthosiphon stamineus* Leaves Extracts

Voucher specimen (no. 11009) of the plant material was deposited at Herbal Room, School of Pharmaceutical Sciences, Universiti Sains Malaysia (USM). WMOS was prepared by having dried and ground *Orthosiphon stamineus* leaves extracted by a mixture of methanol : water (1 : 1) using a Soxhlet extractor for a period of 12 hours, whereas preparation of WOS involved hot maceration of the dried and ground *Orthosiphon stamineus* leaves at 50°C for 6 hours and was repeated thrice. Each extract was bulked and concentrated in a rotary evaporator under vacuum and then freeze-dried and kept in a freezer until used [18]. WOS and WMOS were freshly prepared in distilled water prior to the feeding of the animals.

### 2.2. Animals

Male spontaneously hypertensive rats (SHR, 230–280 g) were housed in individual cages with free access to foods and water and maintained at Animal Transit Facility of School of Pharmaceutical Sciences, USM. All procedures involving animals were conducted according to the ethical guidelines by the Animal Ethics Committee, USM. The animals were divided into four groups: (1) WOS, 1000 mg/kg; (2) WMOS, 1000 mg/kg; (3) losartan, 10 mg/kg; and (4) control (vehicle). All animals were given daily treatment orally for 14 days before being subjected to vascular reactivity studies.

### 2.3. Drugs and Chemicals

Phenylephrine hydrochloride (PE), acetylcholine (ACh), indomethacin, and N^*ω*^-nitro-L-arginine methyl ester (L-NAME) were purchased from Sigma-Aldrich, Germany, while sodium chloride (NaCl), potassium chloride (KCl), potassium dihydrogen phosphate (KH_2_PO_4_), magnesium sulphate (MgSO_4_·7H_2_O), glucose, sodium hydrogen carbonate (NaHCO_3_), and calcium chloride dehydrate (CaCl_2_·H_2_O) were purchased from R&M Chem., UK. All drugs were freshly prepared in normal saline, except indomethacin in 0.5% (w/v) sodium carbonate, prior to use. 

### 2.4. Vascular Reactivity Using Aortic Rings

The rat was anesthetized with sodium pentobarbital (60 mg/kg, i.p.). A midline abdominal incision was performed to expose the aorta. The thoracic aorta was carefully isolated, cleaned from the adherent fat and connective tissues, and cut into 3–5 mm rings. The aortic rings were then suspended horizontally in tissue chambers containing 10 mL of Kreb's solution (mmol/L: NaCl 118.6, KCl 4.8, CaCl_2_ 2.5, MgSO_4_·7H_2_O 1.2, KH_2_PO_4_ 1.2, NaHCO_3_ 25.1, and glucose 11.0). The tissue-bath solution was bubbled incessantly with 95% O_2_ and 5% CO_2_ (carbogen) at 37°C. Aortic rings were then allowed to equilibrate at an optimal tension of 1 g for 45 min. Kreb's solution was replaced every 15 min, and the tension was readjusted to 1 g when necessary. At the beginning of the experiment, the presence of intact endothelial cells was confirmed by precontracting the tissues with PE (1 *µ*M) and followed by relaxation with ACh (1 *μ*M). Relaxation not less than 60% indicated the presence of intact endothelial cells. Responses were recorded isometrically via a force transducer (Grass FT03D) connected to a computerized data acquisition system (PowerLab; ADInstruments Pty Ltd., Australia). For vasoconstriction study, the concentration-response curves for PE (cumulative final chamber concentration of 10^−9^ to 10^−5^ M) were recorded. The contraction effects of PE were recorded in two different preparations, intact and denude endothelium. Denude endothelium of aortic rings was obtained by gently rubbing the intimal layer of the tissue with a blunt needle for a few times. The aortic rings were considered denuded when there were less than 10% relaxations to ACh (1 *μ*M) precontracted with PE (1 *μ*M) whereas in order to obtain the concentration-response curves of relaxation, ACh (10^−9^ to 10^−5^ M) was added cumulatively to the chamber at the plateau of the PE (1 *µ*M) precontracted aortic rings at 3-minute intervals. To further assess the involvement of EDNO and prostacyclin (PGI_2_) releases, relaxations of aortic rings were performed in WOS, WMOS, and losartan groups preincubated for 30 minutes with L-NAME (100 *µ*M), a nonspecific NO synthase inhibitor, and indomethacin (10 *µ*M), a nonselective cyclooxygenase inhibitor, respectively. 

### 2.5. Data Analysis

 All data are given as mean ± standard error means (SEM). PE-induced contraction and ACh-induced relaxation were analysed using one-way ANOVA followed by Dunnett's post hoc test, whereas the effects of ACh-induced relaxation after preincubated by L-NAME and indomethacin were analysed using Student's *t*-test. *E*_max⁡_, *R*_max⁡_, and pD_2_ values were derived from nonlinear regression analysis. All analyses were using the computer software GraphPad Prism 5.0 for Windows (GraphPad Software Inc., USA). Values of *P* < 0.05 were considered statistically significant.

## 3. Results

### 3.1. Vasoconstriction Effects of PE on Aortic Rings

**Figure 1:**
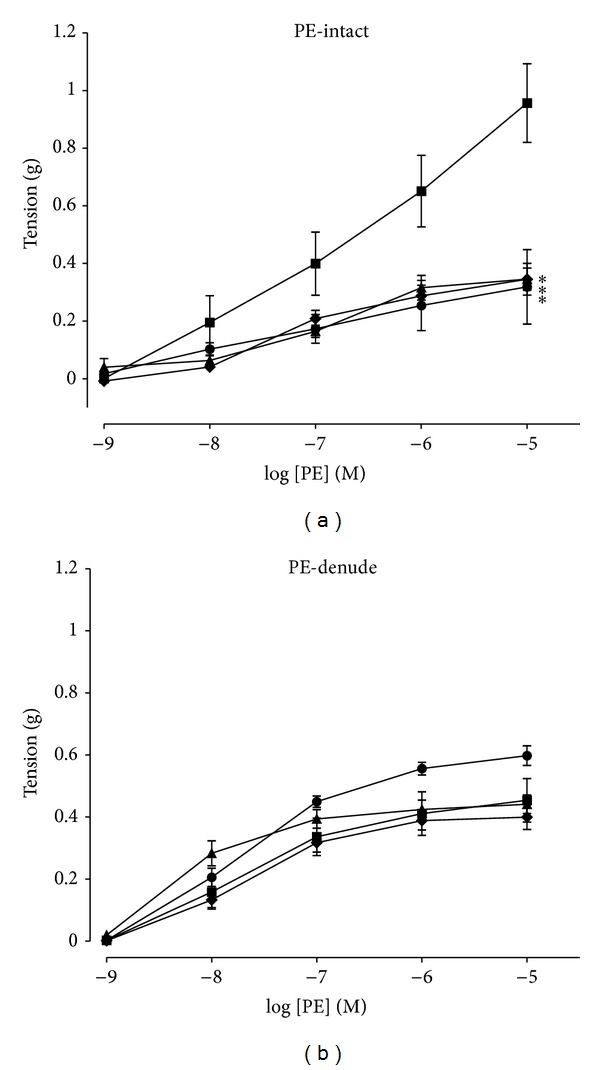
PE-induced contraction responses in intact (a) and denude (b) aortic rings from SHR treated with control (■), losartan (▲), and WOS (●) and WMOS (♦). Responses to PE are expressed as difference between absolute tension developed and baseline tension. Values are mean ± SEM of 5 to 8 SHRs in each group. *Denotes *P* < 0.05 compared to control analyzed by one-way ANOVA followed by Dunnett post hoc test.

Cumulative additions of PE (10^−9^ to 10^−5^ M) produced a concentration-dependent contraction of aortic rings in all groups. In PE intact, WOS, WMOS, and losartan significantly decreased (*P* < 0.05) the contractile responses as compared to control whereas, in PE denude endothelium, no significant changes were obtained (Figure 1). Maximal contractile responses (*E*_max⁡_) in PE intact were significantly decreased in WOS, WMOS, and losartan (0.30 ± 0.06, 0.33 ± 0.03, 0.35 ± 0.03 versus 0.90 ± 0.10). In contrast, the *E*_max⁡_ of WMOS significantly enhanced the contraction responses in PE denude (0.57 ± 0.02 versus 0.43 ± 0.04). The pD_2_ values from both PE intact and denude endothelium were unaltered as shown in Table 1. 

### 3.2. Vasorelaxant Effects of ACh Precontracted with PE on Aortic Rings

**Figure 2:**
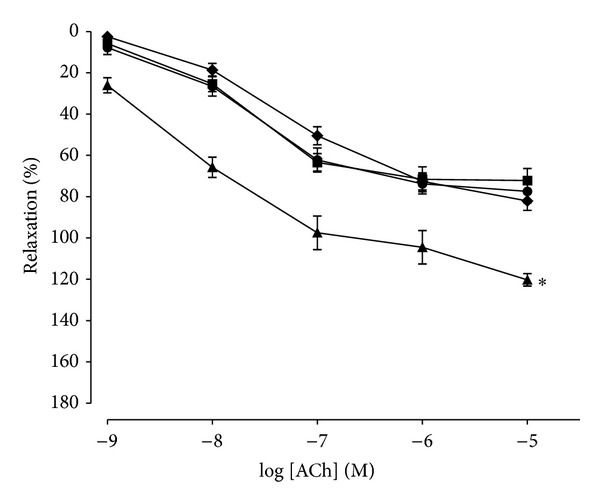
ACh-induced relaxation responses of aortic rings precontracted with PE (1 *µ*M) in control (■), losartan (▲), WOS (●), and WMOS (♦). Responses to ACh are expressed as percentage of relaxation. Values are mean ± SEM of 5 to 8 SHRs in each group. *Denotes *P* < 0.05 compared to control.

ACh (10^−9^ to 10^−5^ M) produced dose-dependent relaxation in all groups in aortic rings precontracted with PE (1 *μ*M). Only losartan significantly enhanced (*P* < 0.05) the relaxant effect of ACh as compared to control (*R*_max⁡_  111.20 ± 4.08 versus 73.15 ± 3.03). Both extract groups did not significantly alter the vasorelaxant effects of ACh as shown in Figure 2 and Table 2.

### 3.3. Effects of L-NAME on ACh-Induced Relaxation in Aortic Rings in WOS, WMOS, and Losartan Groups

**Figure 3:**
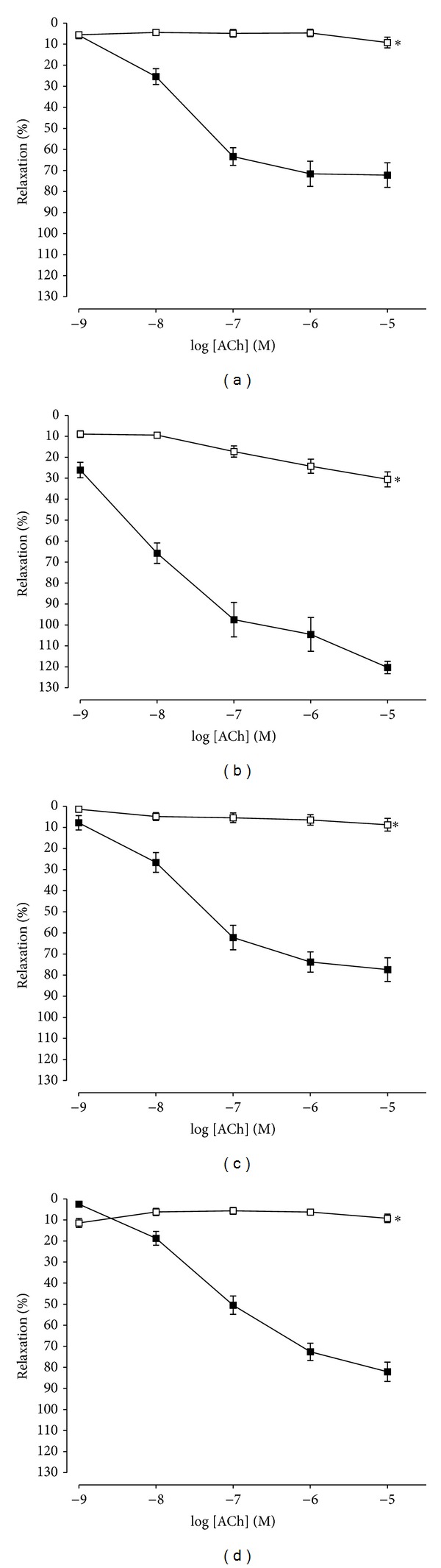
ACh-induced relaxation responses of aortic rings precontracted with PE (1 *µ*M) in the presence of L-NAME (100 *μ*M) in control (vehicle) (a), losartan (10 mg/kg) (b), WOS (1000 mg/kg) (c), and WMOS (1000 mg/kg) (d). Responses to ACh are expressed as percentage of relaxation. Values are mean ± SEM of 5 to 6 SHRs in each group. *Denotes *P* < 0.05 compared to ACh without L-NAME (■).

To assess the contribution of EDNO, the aortic rings were preincubated with L-NAME (100 *µ*M), a NO synthase inhibitor for 30 minutes. ACh-induced relaxations in all groups were significantly inhibited (*P* < 0.05) by L-NAME as shown in Figure 3. *R*_max⁡_ and pD_2_ values were tabulated in Table 2.

### 3.4. Effects of Indomethacin on ACh-Induced Relaxation in Aortic Rings in WOS, WMOS, and Losartan Groups

**Figure 4:**
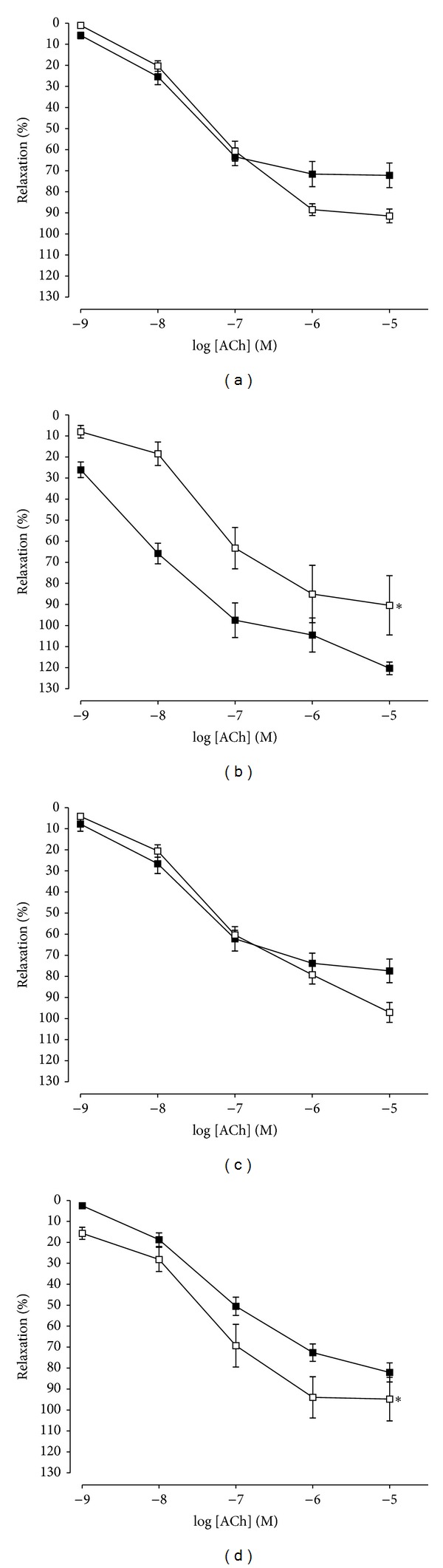
ACh-induced relaxation responses of aortic rings precontracted with PE (1 *µ*M) in the presence of indomethacin (10 *μ*M) in control (vehicle) (a), losartan (10 mg/kg) (b), WOS (1000 mg/kg) (c), and WMOS (1000 mg/kg) (d). Responses to ACh are expressed as percentage of relaxation. Values are mean ± SEM of 5 to 6 SHRs in each group. *Denotes *P* < 0.05 compared to ACh without indomethacin (■).

To investigate the role of prostacyclin (PGI_2_) releases, the aortic rings were preincubated with indomethacin (10 *µ*M), a COX inhibitor for 30 minutes. Indomethacin significantly reduced (*P* < 0.05) the ACh-induced relaxations in losartan and in contrast, significantly improved vasorelaxation in WMOS (Figure 4). *R*_max⁡_ and pD_2_ values were tabulated in Table 2.

### 3.5. Role of Intracellular and Extracellular Calcium Mobilization on the PE-Induced Contraction

**Figure 5:**
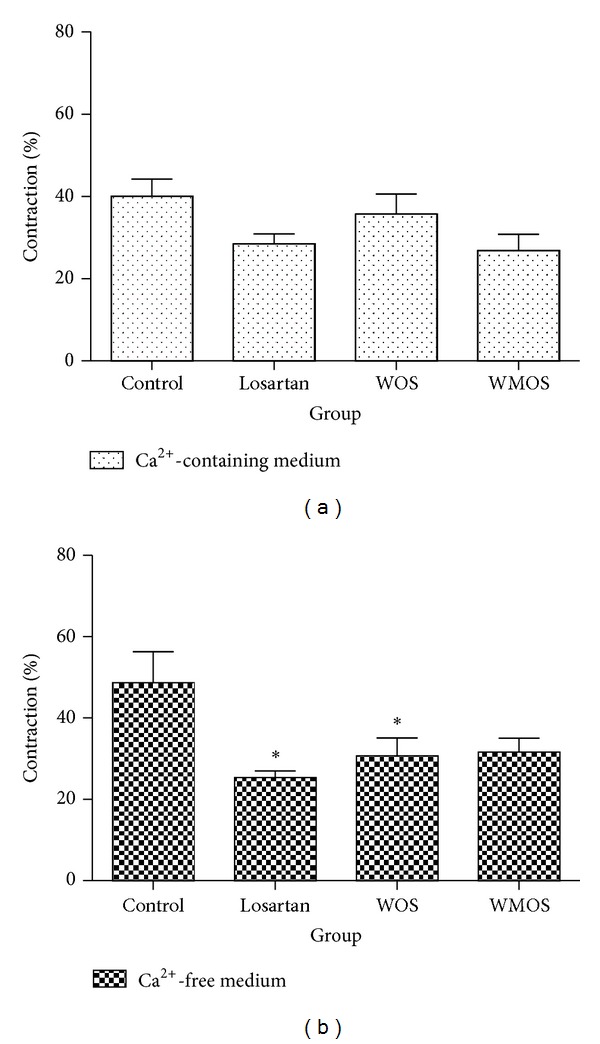
Histograms showing the mean of the response induced by 10^−6^ M PE without endothelium in Ca^2+^-containing medium or Ca^2+^-free medium. Results are expressed as mean ± SEM of 6 experiment sets. *P < 0.05 compared to control.

To assess the role of intracellular and extracellular calcium mobilization, the aortic rings were incubated in Ca^2+^-free medium containing 0.1 mM EGTA. Under this condition, PE induced transient contraction mainly from sarcoplasmic reticulum. In endothelium-denuded aortic rings, a transient contractile response in Ca^2+^-free medium was elicited by 10^−6^ M PE. A second contraction known as sustained contraction was then induced again by PE. The percentage contractile responses to PE were significantly reduced (*P* < 0.05) in losartan (30.69 ± 4.41%) and WOS (25.35 ± 1.61%) as compared to control (48.69 ± 7.59%) in response to PE in Ca^2+^-free medium (Figure 5). When the same procedure was repeated in normal Ca2^+^-containing medium which contained 2.5 mM CaCl_2_, no significant difference was seen in the treatment groups.

## 4. Discussion

The present study demonstrated that, in intact endothelium, the contractile response to phenylephrine (PE), a selective agonist for a_1_-adrenergic receptor, was significantly lowered in SHR treated with WOS and WMOS as compared to control. No significant change was seen in denude endothelium. These results showed that 14-day oral treatment of WOS and WMOS affected the *α*_1_-adrenergic receptors activities in this preparation. The use of PE as a vasoconstrictor in the present study because the rat's aorta has predominantly *α*_1_-postjunctional receptors [14]. Furthermore Griffith et al. [19] and Martin et al. [20] demonstrated that suppression of constrictor responses to several agonists such as PE in the intact vascular endothelium may be due continuously basal release of endothelium-derived relaxing factor (EDRF) from endothelial cells. As seen in the present study, WOS and WMOS inhibited the contraction induced by PE as comparable to losartan. There were studies found that possible crosstalk between AT_1_ and *α*_1_-adrenoceptors existed [21–23]. Furthermore, Maeso et al. [24] reported that losartan reduced vasoconstrictor responses to PE in SHR aortic rings via endogenous Angiotensin II (AngII) acting on AT_1_ receptors. Activation of AT_1_ receptors results in increasing systolic blood pressure (SBP), blood vessels growth, and associated vascular smooth muscle cells (vsmc) apoptosis [25]. Blockade of AT_1_ receptors which inhibit the effects of AngII may promote good prognosis in pathological conditions such as hypertension and to inhibit vasoconstriction [26]. Hence, we may suggest that (1) *Orthosiphon stamineus* leaves extracts exert their antihypertensive effects by blunting the increase of blood pressure in SHR; and (2) *Orthosiphon stamineus* leaves extracts may play their role in reducing vasoconstriction similar as AT_1_ receptor blocker. WOS and WMOS may possibly possess antihypertensive properties and exert similar effects through these interactions. 

Our results showed that the presence of endothelium is very important in vasorelaxation. It is likely that contribution by the variable EDRF such as NO, prostacyclin, and EDHF caused vascular smooth muscle cells to relax. The necessary endothelial cells for the relaxation by acetylcholine (ACh) to be occurred have been discovered since 1980 by Furchgott and Zawadzki. They demonstrated that loss of endothelium by rubbing the intimal surface of aorta caused no relaxation induced by ACh. ACh acts on muscarinic receptors of these cells thus stimulates substances that caused relaxation of vascular smooth muscle cells. In the present study, aortic rings from both WOS and WMOS showed essentially similar relaxant effects with control by dose-response manner to ACh (10^−9^ to 10^−5^ M). It may speculate that both extracts given orally did not alter the endothelium of the rats; thus the aortic rings isolated from these rats showed no effect to the ACh-induced relaxation. In contrast, losartan showed significantly greater relaxation. It is plausible that blockade of AT_1_ receptor further enhanced the relaxation caused by ACh. Furthermore, Schiffrin and Touyz [27] demonstrated that losartan enhanced the endothelium-dependent relaxation to ACh in SHR aortic rings. In this preparation, the relaxation to ACh was probably due to the production or release of EDRF [12] and the endothelial vasorelaxant factors derived from cyclooxygenase (COX) pathways (prostacyclin PGI_2_ released from endothelial cells).

WOS showed similar result of ACh-induced relaxation after preincubation with indomethacin. This similar effect has been reported by Luscher and Vanhoutte [28]. However, WMOS improved vasorelaxation to ACh after blockade of COX pathways. In this case, there was plausibility because the vasodilator PGI_2_ was continuously released as indicated by its tonic effects on platelet cyclic adenosine monophosphate (cAMP) [29]. In contrast, losartan significantly reduced the ACh-induced relaxations, which may be due to the attenuation of PGI_2_ production which was compensated for by the enhanced release of another vasodilator, for example, nitric oxide (NO). In this point of view, we might suggest that WMOS and blockade of AT_1_ receptors modulate the derived endothelial vasorelaxant factors such as PGI_2_ from COX pathways. 

The release of NO by endothelial cells to vascular smooth muscle cells causes vasorelaxation. NO to play a vital role in the maintenance of vascular tone [30]. In order to assess the contribution of NO releases in the vasorelaxant effects elicited by *Orthosiphon stamineus* leaves extracts, we preincubated the rat aortic rings with L-NAME (100 *µ*M), NO synthase inhibitor. Our study showed that the ACh-induced vasorelaxation in all treatment groups reduced significantly after inhibition of NO synthase pathways. From the present data, it could be clearly proven that NO synthase pathways were involved in the vasorelaxation in the SHR. 

In view of the present study, it is plausible that the vasorelaxant activities produced by *Orthosiphon stamineus* extracts may take place in the vascular smooth muscle cells. To investigate the effects of both extracts and losartan on the role intracellular Ca^2+^ on the contractility of the vascular smooth muscle cells of the aortic rings, media absent of and with Ca^2+^ were used. Significantly reduced contraction response to PE in denude aortic rings observed in the WOS and losartan were possibly due to inhibition of intracellular Ca^2+^ release from the sarcoplasmic reticulum at the level of vascular smooth muscle cells. Decreased intracellular Ca^2+^ concentration and increased myosin light chain phosphatase activity may had caused the smooth muscle to undergo weaker vascular contractility. Also, inhibition of receptor- and voltage-operated Ca^2+^ channels in the plasma membrane reduced Ca^2+^ influx may contribute as well [31].

## 5. Summary

In conclusion, our studies showed that water extract (WOS) and water : methanolic (1 : 1) extract (WMOS) of *Orthosiphon stamineus *Benth leaves promote antihypertensive effects by reducing vasoconstriction through the alteration of *α*_1-_adrenergic and AT_1_ receptors activities. Vasorelaxant effects of both WOS and WMOS may possibly involve mainly the release of EDNO. In WOS, PGI_2_ releases might not be participated in the ACh-induced vasorelaxation. However in WMOS, enhancement of vasorelaxation might be due to the fact that vasodilator PGI_2_ is continuously released as indicated by its tonic effects on platelet cAMP. In addition, WOS inhibited the contraction of aortic rings induced by PE, implying that WOS inhibits the release of intracellular Ca^2+^ and/or blocks ROCC.

## Figures and Tables

**Table 1:** Maximal contractile (*E*_max⁡_) responses and sensitivity (pD_2_) for PE intact- and PE denude-induced contraction's in aortic rings.

Group	PE intact		PE denude	
	*E*_max⁡_ (g)	pD_2_ (−log EC_50_)	E_max⁡_ (g)	pD_2_ (−log EC_50_)
Control (vehicle)	0.90 ± 0.10	6.67 ± 0.31	0.43 ± 0.04	7.73 ± 0.32
Losartan (10 mg /kg)	0.35 ± 0.03*	6.84 ± 0.22	0.43 ± 0.02	8.39 ± 0.19
WOS (1000 mg/kg)	0.30 ± 0.06*	7.05 ± 0.62	0.39 ± 0.02	7.72 ± 0.18
WMOS (1000 mg/kg)	0.33 ± 0.03*	7.24 ± 0.22	0.57 ± 0.02*	7.72 ± 0.10

Each value represents the mean ± SEM of 5 to 8 SHRs. *Denotes *P* < 0.05 compared to control for each drug.

**Table 2:** Maximal relaxant effects (*R*_max⁡_) and sensitivity (pD_2_) for ACh-induced relaxation in aortic rings in the absence and presence of indomethacin and L-NAME.

Treatment groups	ACh		ACh + indomethacin		ACh + L-NAME	
	*R*_max⁡_ (% of relaxation)	pD_2_ (−log EC_50_)	*R*_max⁡_ (% of relaxation)	pD_2_ (−log EC_50_)	*R*_max⁡_ (% of relaxation)	pD_2_ (−log EC_50_)
Control	73.15 ± 3.03	7.72 ± 0.15	91.74 ± 2.39	7.33 ± 0.08	NI	NI
Losartan (10 mg/kg)	111.20 ± 4.08^#^	7.98 ± 0.17	90.20 ± 7.64*	7.31 ± 0.28	29.55 ± 2.46*	6.73 ± 0.30
WOS (1000 mg/kg)	76.42 ± 3.39	7.62 ± 0.17	91.04 ± 3.07	7.26 ± 0.11	7.12 ± 1.35*	8.17 ± 0.88
WMOS (1000 mg/kg)	79.56 ± 2.97	7.24 ± 0.12	96.37 ± 6.33*	7.32 ± 0.24	NI	NI

Values are mean ± SEM of 5 to 8 SHRs in each group. ^#^Denotes *P* < 0.05 compared to control and *denotes *P* < 0.05 compared to ACh without inhibitors. NI: not identified by nonlinear regression analysis by GraphPad Prism 5.0.
